# Microwave Radiation Remodels Hippocampal Astrocytes Subpopulations and Intercellular Communication at Single-Cell Resolution

**DOI:** 10.3390/cells15121121

**Published:** 2026-06-22

**Authors:** Chenxu Chang, Zhihua Feng, Yumeng Ye, Zhengtao Xu, Xiaoxu Kong, Ying Liu, Xuelong Zhao, Yanhui Hao, Hongyan Zuo, Yang Li

**Affiliations:** Beijing Institute of Radiation Medicine, Beijing 100850, China

**Keywords:** hippocampus, astrocyte, cognitive impairment, microwave radiation, single-cell RNA sequencing

## Abstract

The potential health hazards caused by microwave exposure have attracted increasing attention. Microwave radiation has been reported to induce oxidative stress in neural tissues, which is considered one of the primary mechanisms underlying its adverse effects on central nervous system function. The hippocampus is sensitive to microwave radiation, whereas underlying cellular and molecular mechanisms remain incompletely understood. In this study, microwave-exposed mice exhibited significantly impaired performance in the Go/No-go, Y-maze, and novel object recognition tests at 6 h and 7 days post-exposure, indicating deficits in hippocampus-dependent working memory. Single-cell RNA sequencing of hippocampal tissues from control and microwave-exposed mice yielded 94,088 high-quality cells across eight major cell types. Astrocyte sub-clustering identified five transcriptionally distinct subpopulations, with Astrocyte_S100a6 and Astrocyte_Son proportions increased and Astrocyte_Serpinf1 decreased in the radiation group. Analysis of astrocyte transcriptional state transitions showed microwave-exposed astrocytes were preferentially distributed toward terminal reactive states with depletion at early homeostatic nodes. Cell–cell communication analysis revealed increased total interactions and interaction strength following radiation. Astrocyte outgoing signaling was increased for pathways associated with vascular remodeling, phagocytic regulation, and neuroinflammation, while pathways related to trophic support were decreased. Incoming signaling showed increased activity in pathways linked to phagocytic recruitment and inflammatory mediation. Taken together, these findings indicate that microwave exposure is associated with hippocampus-dependent working memory deficits accompanied by transcriptional remodeling of astrocyte subpopulation composition, directional astrocyte state transitions toward reactive phenotypes, and broad alterations in astrocyte-centered intercellular communication, providing a cellular and molecular framework for understanding astrocyte involvement in microwave radiation-associated hippocampal dysfunction.

## 1. Introduction

The growing prevalence of electronic devices and communication systems in daily life has raised public health concerns. Electromagnetic radiation is ubiquitous in the environment. Microwaves are a form of electromagnetic waves with wavelengths ranging from 1 mm to 1 m (corresponding to frequencies between 300 MHz and 300 GHz). Microwaves are widely used, and microwave radiation (MR) is emitted by various communication facilities. In addition, microwaves are extensively applied in organic synthesis and food processing owing to their ability to deliver rapid volumetric heating. However, this heating mode can cause non-uniform temperature distributions and localized overheating, which may promote the thermal degradation of heat-labile food additives and generate potentially hazardous byproducts. For example, thermal degradation of sucralose, a widely used synthetic trichlorinated high-intensity artificial sweetener, has been demonstrated to produce a range of chlorinated compounds [1,2]. Epidemiological studies suggest that long-term exposure to MR may adversely affect multiple physiological systems, including the immune, nervous, cardiovascular, and reproductive systems [3,4,5]. For instance, MR has been linked to immune suppression, reduced infection resistance, and increased cancer risk, as well as neurological disturbances, abnormal electrocardiograms, and cataracts. The underlying mechanisms of these effects include oxidative stress, altered gene expression, disrupted cellular signaling pathways, etc. [6,7,8]. However, the molecular mechanisms of MR remain incompletely elucidated, especially because of uncertainty regarding which cell types are sensitive to MR. Therefore, further studies based on novel biotechnologies are essential to better understand the effects of MR and develop effective mitigation strategies.

Oxidative stress is considered a primary mechanism underlying the biological effects of MR at the cellular level. Upon microwave exposure, excessive reactive oxygen species (ROS) are generated through mitochondrial electron transport chain dysfunction, leading to lipid peroxidation of cellular membranes, protein carbonylation, and even single- and double-strand DNA breaks. At the molecular level, MR-induced ROS impair the endogenous antioxidant defense system, downregulating key enzymes including superoxide dismutase (SOD), catalase (CAT), and glutathione peroxidase (GPx), thereby amplifying oxidative damage [9,10]. Downstream consequences include activation of pro-apoptotic pathways via cytochrome c release and caspase-3 activation, dysregulation of mitogen-activated protein kinase (MAPK) cascades, and upregulation of nuclear factor kappa B (NF-κB)-mediated inflammatory signaling, collectively contributing to cellular dysfunction and death [11].

**Figure 1:**
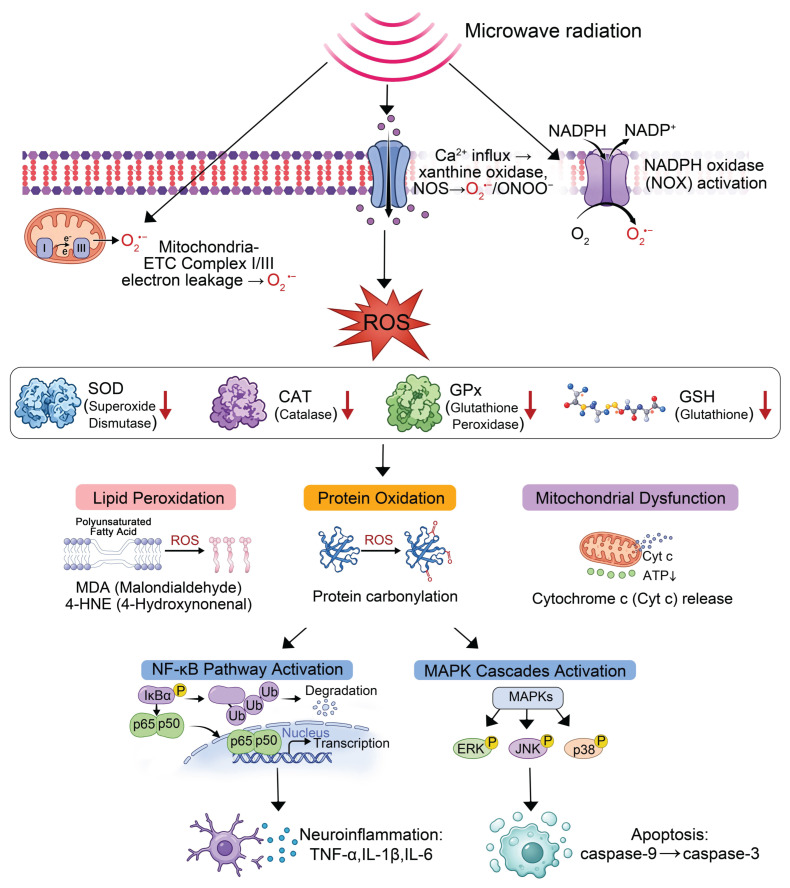
Microwave radiation induces oxidative stress and downstream cellular sequelae. Red downward arrows indicate decreases in the levels of SOD, CAT, GPx, and GSH.

Excessive oxidative stress triggers a cascade of downstream biological effects that ultimately lead to neuronal dysfunction and cognitive impairment. ROS-mediated lipid peroxidation disrupts cellular membrane integrity, while protein oxidation impairs enzyme activity and structural protein function. Oxidative DNA damage, particularly mtDNA mutations, further exacerbates mitochondrial dysfunction [12]. Additionally, ROS act as a second messenger to activate redox-sensitive transcription factors, which upregulates the expression of pro-inflammatory cytokines [13]. This neuroinflammatory response drives the activation of astrocytes and microglia, which in turn produce additional ROS and pro-inflammatory mediators, creating a self-sustaining cycle of neuroinflammation and oxidative damage [14]. Notably, astrocytes, as the most abundant glial cells in the brain, play a central role in this process by regulating neuronal redox homeostasis and synaptic function, making them particularly susceptible to MR-induced oxidative stress (Figure 1).

In addition to acute oxidative damage, MR induces persistent alterations in gene expression through epigenetic mechanisms. MR has been associated with aberrant DNA methylation patterns, including hypermethylation at promoter regions of tumor suppressor genes mediated by DNMT1 and DNMT3A, and reduced activity of the demethylase TET2. Histone modification changes, including altered H3K4me3 (active transcription mark) and H3K27me3 (repressive mark) distributions, have been observed following MR, affecting the transcriptional regulation of stress-response and inflammatory genes [15]. Furthermore, dysregulation of non-coding RNAs, including upregulation of miR-21 and miR-34a and altered expression of lncRNA *MALAT1*, has been reported in MR-exposed neural cells, suggesting additional layers of post-transcriptional regulation [16].

The nervous system is particularly sensitive to MR for several reasons. First, neural cell membranes contain an exceptionally high proportion of polyunsaturated fatty acids, rendering them highly susceptible to ROS-mediated lipid peroxidation [17]. Second, the brain exhibits the highest metabolic rate and oxygen consumption of any organ, resulting in elevated baseline ROS production and reduced capacity to buffer additional oxidative insults [18]. Third, mature neurons possess limited regenerative capacity, meaning that radiation-induced damage may have lasting functional consequences. Fourth, the brain’s relatively low expression of antioxidant enzymes compared to peripheral tissues further diminishes its capacity for oxidative stress compensation [19]. Collectively, these factors render the brain tissue particularly vulnerable to MR-associated dysfunction, consistent with multiple reports linking MR exposure to hippocampus-dependent cognitive impairment. While previous studies have reported transcriptional changes in select genes within hippocampal tissue following MR, few have investigated cell-type-specific contributions to MR-induced hippocampal dysfunction [20,21,22,23].

Among the various cell types in the central nervous system (CNS), astrocytes are especially abundant and play essential roles in maintaining CNS homeostasis. They interact with neurons, glial cells, and blood vessels, support CNS structure, axon guidance, synapse creation, synaptic transmission modulation, metabolism, blood–brain barrier development, and vascular blood flow regulation [24,25,26]. Astrocytes serve as the primary interface between the vasculature and neurons, are key mediators of neuroinflammation and oxidative stress responses. Importantly, microwave exposure induces alterations in multiple signaling molecule receptors in astrocytes, such as purinergic receptors and transient receptor potential (TRP) channels [27,28]. Furthermore, accumulating evidence suggests that astrocyte dysfunction precedes and contributes to neuronal damage in multiple CNS injury models, making them a particularly relevant cellular target for investigating MR-associated hippocampal dysfunction [29,30].

Prior transcriptomic studies have provided foundational characterizations of astrocyte gene expression in health and disease. A previous study identified distinct reactive astrocyte subtypes, termed A1 (neurotoxic) and A2 (neuroprotective), each with characteristic gene expression signatures in response to CNS injury [31]. Subsequent single-cell RNA sequencing studies revealed that astrocytes comprise multiple transcriptionally distinct subpopulations with region-specific and disease-specific properties, substantially revising the earlier binary classification framework [32,33,34]. However, cell-type-specific transcriptional responses of astrocytes to MR, and the extent to which known reactive astrocyte states are engaged following radiation exposure, remain largely uncharacterized.

Recent studies have revealed the crucial role of astrocytes in learning and memory [35,36,37,38]. Moreover, behavioral disorders such as cognitive dysfunction caused by MR have been demonstrated in both human and animal studies. Although few studies have examined the effect of electromagnetic radiation on astrocytes, accumulating evidence suggests that astrocytes may be important in MR-induced cognitive dysfunction. This study explored the cell-type-specific transcriptional alterations and mechanism underlying MR-induced hippocampal dysfunction. Using single-cell RNA sequencing (scRNA-seq), we characterized astrocytes-specific changes and identified differentially expressed genes at the single cell level in the hippocampus after microwave exposure. In addition, this study also investigated the role of hippocampal astrocytes in mediating MR-induced cognitive dysfunction.

## 2. Materials and Methods

### 2.1. Animals

C57BL/6N male and female mice (8 weeks of age) were provided by the Beijing Vital River Company (Beijing, China). All mice were reared in a specific pathogen-free (SPF) animal room at 20–26 °C and 40–60% humidity under a 12 h light/dark cycle. The mice were randomly divided into control (Con) and MR groups. All animal experiments reported in this study were conducted according to the experimental protocol approved by the Institutional Animal Care and Use Committee of Academy of Military Medical Sciences (No. IACUC-DWZX-2021-685).

### 2.2. Microwave Radiation

**Figure 2:**
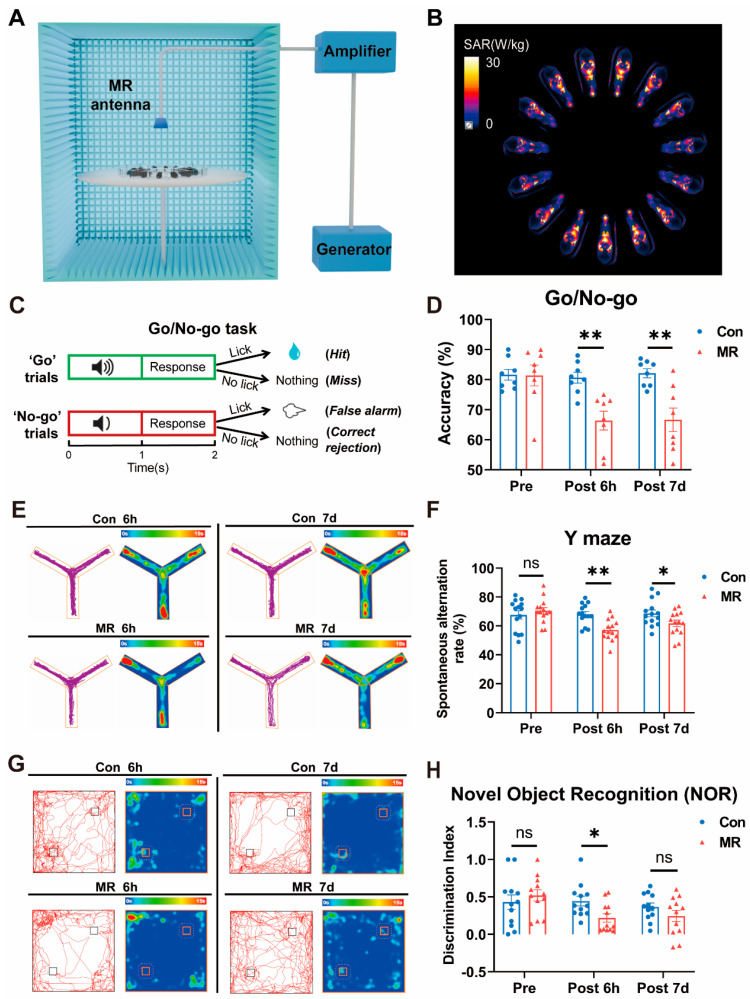
Schematic diagram of the microwave exposure system and effects of microwave exposure on hippocampus-dependent working memory in mice. (**A**) Schematic diagram of the microwave exposure system; (**B**) SAR map of Diggy Male Nude Normal Mouse Model simulated using Sim4Life 6.2 software; (**C**,**D**) Go/No-go tasks (*n* = 8 mice). A schematic of the Go/No-go task (**C**) and changes in accuracy percentages before and after microwave exposure (**D**). (**E**,**F**) Y-maze tests (*n* = 14 mice). Heat map of the total motion (**E**) and spontaneous alternation rates (%) (**F**) for each group. (**G**,**H**) NOR tests (*n* = 12 mice). Representative movement trace of individual animal in each group (**G**), novel and familiar objects are represented by small boxes. Discrimination index of each group in the NOR tests (**H**). SAR, specific absorption rate; NOR, novel object recognition. Data are presented as mean ± SEM. Statistical analyses were performed using two-tailed Student’s *t*-test (**D**,**F**,**H**). *, *p* < 0.05; **, *p* < 0.01; ns, non-significant (*p* > 0.05).

The microwave exposure system was as previously described [39]. Briefly, the microwave source with a central frequency of 2856 MHz was placed in an electromagnetic shield chamber (GuoruiZhaofu Electronic, Wuhu, China). The mice in the MR group were placed in a transparent container of porous plexiglass with vent holes, which was placed on a circular platform below the microwave antenna (Figure 2A). The distance between the microwave antenna and the dorsal surface of the mice was fixed at 127 cm. During the radiation, the platform was rotated to ensure uniform radiation dose distribution in the mice. MR was applied for 15 min an average power density of 8 mW/cm^2^, a repetition frequency of 80 Hz, and a duty cycle of 4%. The mice in the Con group were subjected to pseudo radiation under identical conditions. A specific absorption rate (SAR) map was generated using Sim4Life v6.2 (ZMT, Switzerland) based on the Diggy Male Nude Normal Mouse with a body weight of 23.3 g (Figure 2B).

### 2.3. Animal Behavioral Tests

#### 2.3.1. Go/No-Go Task

The mice were trained using a sound-dependent Go/No-go task (ThinkerTech Nanjing Bioscience Inc., Nanjing, China). Two days prior to behavioral training, all mice were placed on a controlled water restriction protocol with a fixed daily ration of 2.0 mL per mouse. Water restriction was maintained throughout the entire experimental period to maintain each animal’s body weight at 85–90% of its pre-restriction ad libitum baseline weight. The mice received a water reward by licking a water spout during the training and test procedures. Once the mice reliably licked the spout (2–3 days), they were subjected to the Go/No-go training. One hundred trials were conducted daily. For the Go trials, a sound (5000 Hz) stimulus cue with a 3 s duration was delivered, followed by a response window (3 s). Licking during the response window was rewarded with a drop of water. For the No-go trials, a different sound stimulus cue (10,000 Hz) with a 3 s duration was delivered, followed by a response window (3 s). Licking during the No-go response window was punished by a foot shock. The Go and No-go trials were randomly interleaved. A correct response in the Go trial (hit) occurred when the mouse successfully licked the spout during the response window and subsequently received a water reward. A correct response in the No-go trial (correct rejection) occurred when the mouse successfully withheld the lick response during the response window, thus avoiding shock. The overall performance was calculated as the total number of correct responses divided by the total number of trials: overall performance = (hits + correct rejects)/(total trials) (Figure 2C).

#### 2.3.2. Y-Maze

After environmental adaptation in the experimental room for 30 min per day for three-consecutive days, the mice were placed in a symmetrical three-arm Y-maze made of gray plastic (Shenzhen RWD Life Science Co., Ltd., Shenzhen, China). The three arms were identical, with 120° angles. Each arm was 30 cm long, 8 cm wide, and 15 cm high. The mice were randomly placed in one of the arms and allowed to explore freely for 8 min. The movement traces were recorded and analyzed using ANY-maze (version 7.00) behavioral tracking software (Stoelting Co., Wood Dale, IL, USA). Spontaneous alternations were measured, and mouse exploration was evaluated. Perfect alternations were defined as the sequential exploration of all three arms sequentially before re-entering a previously visited arm. Entrances were defined as the presence of four paws within the arm. The results are presented as the ratio of the number of perfect alternations to the total number of possible alternations.

#### 2.3.3. Novel Object Recognition (NOR)

For environmental adaptation, the mice were kept in the experimental room for at least 30 min per day for three consecutive days. The mice were then placed in an open-field box (40 cm side length) with two identical objects and allow to explore freely for 5 min (Shenzhen RWD Life Science Co., Ltd., Shenzhen, China). After a one-hour inter-trial interval, the mice were placed back in the open-field box, with one familiar object and one novel object at the same position, and again allowed to explore freely for another 5 min. Movement traces were generated using ANY-maze (version 7.00) behavioral tracking software (Stoelting Co., Wood Dale, IL, USA). The discrimination index was determined using the exploration time of objects according to the following formula: (TNovel − TFamiliar)/(TNovel + TFamiliar).

### 2.4. scRNA-Seq

#### 2.4.1. Preparation of Single-Cell Suspension of Mouse Hippocampus

Mouse hippocampal tissues were dissociated into single-cell suspensions using the Adult Brain Dissociation Kit (Cat. No. 130-107-677, Miltenyi Biotec GmbH, Bergisch Gladbach, Germany). Briefly, mice were deeply anesthetized, brains were rapidly removed, and bilateral hippocampal tissues were immediately dissected on ice and minced into small pieces. Tissue pieces were transferred to a gentleMACS C Tube containing Enzyme Mix 1 (Enzyme P reconstituted in Buffer Z) and subjected to the pre-programmed “37C_ABDK_01” dissociation run on a gentleMACS™ Octo Dissociator with Heaters (Miltenyi Biotec). Enzyme Mix 2 (Enzyme A in Buffer Y) was then added and the program was repeated. The homogenate was filtered through a 70 µm cell strainer, and myelin debris was removed using the Debris Removal Solution, followed by erythrocyte lysis with the Red Blood Cell Removal Solution. The purified pellet was resuspended in phosphate-buffered saline (PBS; Gibco, Thermo Fisher Scientific, Waltham, MA, USA) containing 0.04% bovine serum albumin (BSA; Thermo Fisher Scientific, Waltham, MA, USA). Cell viability was assessed by trypan blue exclusion (Gibco, Thermo Fisher Scientific, Waltham, MA, USA) using a hemocytometer, and only samples with viability ≥ 85% and a concentration of 700–1200 cells/μL were used for scRNA-seq.

#### 2.4.2. Single-Cell RNA Library Preparation and Sequencing

Single-cell RNA libraries were prepared using the 10x Genomics Chromium Single Cell 3’ Reagent Kit (v3.1) (Thermo Fisher Scientific, Waltham, MA, USA) according to the manufacturer’s instructions. Briefly, single-cell suspensions were loaded onto the Chromium Controller to generate gel bead-in-emulsion (GEM) droplets, targeting a recovery of approximately 10,000 cells per sample. Cell barcoding, reverse transcription, and cDNA amplification were performed within GEMs. Sequencing libraries were constructed following cDNA amplification, end repair, A-tailing, adapter ligation, and sample index PCR. Library quality and concentration were assessed using an Agilent Bioanalyzer 2100 (Agilent Technologies, Santa Clara, CA, USA) and a Qubit fluorometer (Thermo Fisher Scientific, Waltham, MA, USA), respectively. Sequencing was performed on an Illumina NovaSeq 6000 platform (Illumina Inc., San Diego, CA, USA) with paired-end 150 bp reads, targeting a minimum sequencing depth of 20,000 reads per cell.

#### 2.4.3. Single-Cell Gene Expression Quantification and Quality Control

Raw sequencing reads were aligned to the mouse reference genome (GRCm38/mm10) and gene expression matrices were generated using Cell Ranger with default parameters. For quality control, cells were filtered based on the following criteria: number of detected genes per cell between 200 and 6000, total UMI counts per cell between 500 and 30,000, and mitochondrial gene expression fraction less than 10%. Genes detected in fewer than three cells were excluded from downstream analysis. After quality control, filtered gene expression matrices were imported into Seurat [40] for subsequent analysis. Gene expression data were normalized using the NormalizeData function with a scale factor of 10,000, followed by log-transformation.

#### 2.4.4. Cell-Type Clustering and Marker Identification

Following quality control, highly variable genes (HVGs) were identified using the FindVariableFeatures function (nfeatures = 2000). Gene expression data were scaled and principal component analysis (PCA) was performed using the top 2000 HVGs. Neighborhood graphs were constructed using the FindNeighbors function (dims = 1:30), and unsupervised clustering was performed using the Louvain algorithm implemented in FindClusters with a resolution of 1.0. Dimensionality reduction for visualization was performed using UMAP. Cluster marker genes were identified using the FindAllMarkers function with the Wilcoxon rank-sum test, retaining genes with log2 fold change > 0.25 and adjusted *p*-value < 0.05 expressed in at least 25% of cells within the cluster. Cell-type identity was manually annotated based on the expression of established canonical marker genes for each major brain cell type.

#### 2.4.5. Cell Type Identification

A final dataset of 94,088 individual cells was preserved to map the landscape of hippocampus and roughly classified into microglia, oligodendrocytes, astrocytes, oligodendrocyte progenitor cells (OPCs), neurons, endothelial cells, pericytes and ependymal cells. For subclustering, astrocytes were separately subsetted and reclustered with an optimal resolution based on the number of cells according to the Seurat guidelines.

#### 2.4.6. Astrocyte Subpopulation Identification and Characterization

Astrocytes were extracted and re-clustered using the FindNeighbors and FindClusters functions with a resolution of 1.0, yielding 11 transcriptionally distinct clusters visualized by UMAP. Cluster annotation was performed based on the expression of canonical marker genes, and clusters were merged into five biologically defined subpopulations: Ast_Slc7a10, Ast_S100a6, Ast_Son, Ast_Serpinf1, and Ast_Tpt1. Marker genes for each subpopulation were identified using the FindAllMarkers function with the Wilcoxon rank-sum test. Subpopulation-specific marker gene expression was visualized as dot plots showing average expression and percentage of expressing cells. The proportional composition of astrocyte subpopulations across experimental groups (Con and MR) and individual samples was summarized as stacked bar plots.

#### 2.4.7. Pseudotime Trajectory Analysis

Pseudotime trajectory analysis of astrocyte subpopulations was performed using Monocle 2 [41,42]. Astrocytes were extracted from the integrated Seurat object and imported into Monocle 2. Highly variable genes were selected as ordering genes based on mean expression and dispersion thresholds. Dimensionality reduction was performed using the DDRTree algorithm, and cells were ordered along the reconstructed trajectory using the orderCells function. The root state was defined based on the expression profile consistent with a homeostatic astrocyte identity. Trajectory plots were colored by transcriptional state, pseudotime value, cell subpopulation identity, and experimental group (Con vs. MR), respectively.

#### 2.4.8. Cell–Cell Communication Analysis

Intercellular communication networks were inferred and compared between the Con and MR groups using CellChat [43]. Each group was analyzed independently using the mouse CellChatDB ligand-receptor database. Communication probabilities were computed at both the ligand-receptor and signaling pathway levels, and aggregate interaction numbers and weights were calculated for each directed cell-type pair. Outgoing and incoming signaling patterns for each cell type were visualized as heatmaps based on relative signaling strength. Circle plots were used to illustrate the global communication network topology for each group.

### 2.5. Statistical Analysis

All statistical analyses and graph generation were performed using R software (version 4.3.3) and GraphPad Prism (version 8.0). Statistical analysis of the differences between the two groups was performed using a two-tailed Student’s *t*-test. For all statistically significant indications in this study, **, *p* < 0.01; *, *p* < 0.05; and ns, not significant.

## 3. Results

### 3.1. Impaired Hippocampus-Dependent Working Memory After Microwave Exposure

The hippocampus is an essential component of cognitive functions, including learning, memory, and emotion regulation. To further investigate the effects of microwave exposure on hippocampus-dependent working memory, we performed the Go/No-go task, Y maze and NOR behavioral test. First, the Go/No-go task was designed to train the conditioned reflexes of the animals and analyze their ability to discriminate among sounds. The mice were trained to discriminate between two types of sounds with different frequencies of 5000 Hz and 10,000 Hz, respectively, to receive a water reward. The mice learned to lick the tube during the Go trial (5000 Hz) and did not lick the tube during the No-go trial (10,000 Hz). The possible behavioral responses included hit, miss, correct rejection (CR) and false alarm (FA) (Figure 2C). The results significantly decreased the performance of mice at 6 h and 7 d post-radiation compared with the Con group (*p* < 0.05), indicating that microwave exposure impaired reward working memory in mice (Figure 2D).

The Y-maze is primarily used to study spatial working memory in rodents. Owing to the nature of experimental animals, which involves exploring new environments and remembering the previous exploration direction every time they change exploration directions. Thus, the Y-maze experiment can effectively measure the ability of spatial working memory in animals. For the Y-maze test, the mice were randomly placed in one of the arms and allowed to explore freely for 8 min. The results showed significantly decreased alternation of arm entries in the MR group compared with the Con group at 6 h and 7 d after microwave exposure (*p* < 0.05), whereas no significant difference was observed between the two experimental groups before microwave exposure (Figure 2E,F). These results further suggest that microwave exposure leads to a decline in spatial working memory in mice.

The NOR experiment was based on the tendency of rodents to explore new objects, with characteristics of short cycles, learning, and memory testing in a state of free activity without stimulation. NOR is widely used to evaluate recognition and memory abilities in mice. In this study, mice in the MR group showed a reduced discrimination index compared with those in the Con group (*p* < 0.05) at 6 h after microwave exposure (Figure 2G,H). These findings indicate that microwave exposure inhibits novelty-seeking behavior in mice. Consistent with the Go/No-go and Y-maze test results, the NOR test findings suggested that microwave exposure induces hippocampal-dependent working memory in mice.

### 3.2. Single-Cell Transcriptome Profiling of the Hippocampus After Microwave Exposure

**Figure 3:**
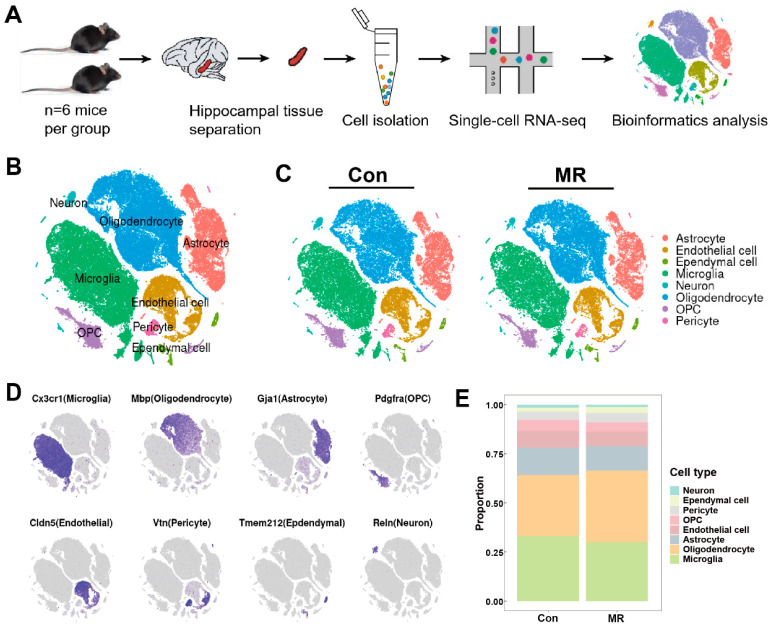
Classification of cell types in the mouse hippocampus based on scRNA-seq data after microwave exposure. (**A**) Schematic workflow of scRNA-seq using hippocampus samples from the Con and MR groups (*n* = 6); (**B**) t-SNE plot showing eight major cell types in the hippocampus based on transcriptomic data; (**C**) t-SNE plots showing eight cell types in the Con and MR groups; (**D**) Expression of marker genes (highlighted in purple) for eight cell types in the t-SNE plots; (**E**) Proportions of the eight cell types in the Con and MR groups.

Understanding the cell types and molecular composition of the hippocampus is important for further elucidating the memory mechanisms. scRNA-seq technique has opened new possibilities for identifying such cell type-specific molecular changes, as well as disease-specific cell states, and examining molecular mechanisms in the hippocampus. We performed transcriptome analysis of 12 independent biological samples (six sham samples and six radiation samples) at the single-cell level using scRNA-seq at 1 d after microwave exposure to investigate how the molecular and cellular profiles of hippocampus are altered after microwave exposure compared to those in healthy subjects (Figure 3A). After filtering out the low-quality cells, 94,088 high-quality cells were obtained and separated into eight cell types (Figure 3B,C). According to the expression patterns of cell type-specific marker genes, the neural cells were annotated as astrocytes (*Gja1*), microglia (*Cx3cr1*), oligodendrocytes (*Mbp*), oligodendrocyte precursor cells (OPCs) (*Pdgfra*), neurons (Reln), non-neural cells including endothelial cells (*Cldn5*), pericytes (*Vtn*), and ependymal cells (*Tmem212*) (Figure 3D). Comparison of the proportions of these cell types between the control and radiation samples revealed similar proportions (Figure 3E).

### 3.3. Identification of Five Transcriptionally Distinct Astrocyte Subpopulations

**Figure 4:**
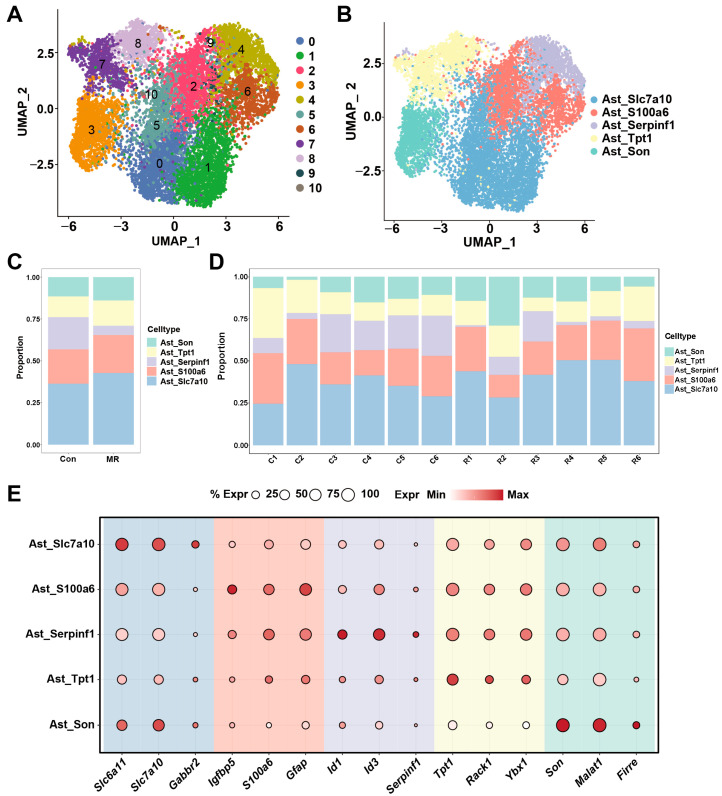
Single-cell transcriptomic profiling of astrocyte subpopulations in the hippocampus after microwave exposure. (**A**) UMAP plot of all astrocytes colored by unsupervised clustering, yielding 11 distinct clusters; (**B**) UMAP plot with cells colored by annotated subpopulation identity (Ast_Slc7a10, Ast_S100a6, Ast_Son, Ast_Serpinf1, and Ast_Tpt1); (**C**) Stacked bar plot showing the proportional composition of astrocyte subpopulations in the Con and MR groups; (**D**) Sample-level stacked bar plots depicting subpopulation proportions across individual Con (C1–C6) and MR (R1–R6) samples; (**E**) Dot plot illustrating the average expression (color intensity) and percentage of expressing cells (dot size) for subpopulation marker genes across the five astrocyte subtypes.

To characterize the heterogeneity of astrocytes at single-cell resolution, we performed unsupervised clustering of all astrocyte-lineage cells, which yielded 11 transcriptionally distinct clusters (clusters 0–10) visualized by Uniform Manifold Approximation and Projection (UMAP) (Figure 4A). Based on the expression of canonical marker genes, these initial clusters were subsequently annotated into five biologically defined astrocyte subpopulations: Ast_Slc7a10, Ast_S100a6, Ast_Son, Ast_Serpinf1, and Ast_Tpt1 (Figure 4B).

The identity of each subpopulation was supported by a curated panel of 17 marker genes examined via dot plot analysis (Figure 4E). Ast_Slc7a10 showed prominent expression of *Slc6a11*, *Slc7a10*, and *Gria2*, consistent with a homeostatic, glutamate-metabolism-associated astrocyte phenotype. Ast_S100a6 was characterized by high expression of *S100a6*, *Igfbp5*, and *Gfap*, suggesting a reactive or stress-responsive identity. Ast_Son was distinguished by strong expression of *Son* and *Malat1*, implicating active transcriptional and RNA-processing functions. Ast_Serpinf1 showed elevated expression of *Id1*, *Id3*, and *Serpinf1*, genes associated with cell fate regulation and neuroprotective secretion. Ast_Tpt1 was marked by high expression of *Tpt1*, *Rack1*, and *Ybx1*, indicating roles in translational control and cellular stress responses.

To assess potential MR-associated compositional shifts, we compared the proportional distribution of these five subpopulations between the Con and MR groups (Figure 4C). The MR group exhibited a notable increase in the proportion of Ast_S100a6 and Ast_Son, accompanied by a relative decrease in Ast_Serpinf1, suggesting a shift from homeostatic toward reactive astrocyte states under pathological conditions. This compositional change was further examined at the individual sample level across six Con samples (C1–C6) and six MR samples (R1–R6), which revealed consistent inter-sample trends while also highlighting a degree of inter-individual variability within each group (Figure 4D). Together, these data demonstrate the transcriptional diversity of astrocytes and reveal a MR-associated remodeling of the astrocyte subpopulation landscape.

### 3.4. Pseudotime Trajectory Analysis Reveals Divergent Astrocyte Differentiation Dynamics in MR Conditions

**Figure 5:**
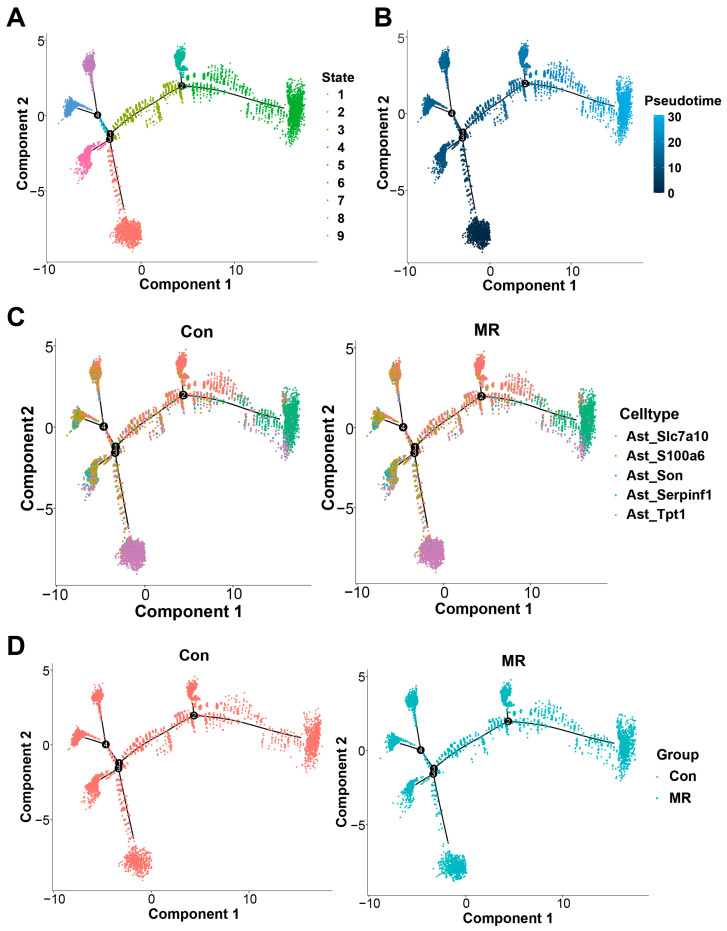
Pseudotime trajectory analysis of astrocyte subpopulations. (**A**) Monocle-based pseudotime trajectory of all astrocytes colored by transcriptional state (States 1–9), with four branch nodes labeled (1–4); (**B**) The same trajectory colored by pseudotime value (0–30), with darker blue indicating earlier pseudotime and lighter blue indicating later developmental stages; (**C**) Split trajectory plots comparing the Con and MR groups, with cells colored by astrocyte subpopulation identity; (**D**) Overlay trajectory plot with cells colored by experimental group (Con, pink; MR, teal), illustrating group-specific differences in trajectory distribution and terminal branch occupancy.

To reconstruct the developmental trajectory and state transition dynamics of astrocytes, we performed pseudotime analysis using Monocle on the five annotated subpopulations. The resulting trajectory resolved into nine transcriptional states (States 1–9) connected through four principal branch nodes (nodes 1–4), forming a multi-branching topology that captures distinct differentiation paths (Figure 5A). The trajectory backbone comprised a primary axis extending along Component 1, with lateral branches emanating at nodes 3 and 4, indicating the existence of at least three major terminal cell fates.

Pseudotime ordering assigned the earliest developmental stage (pseudotime ≈ 0) to cells concentrated near nodes 1 and 3 at the lower-left region of the trajectory, with progressive maturation values increasing along the main axis toward the right terminal end and through diverging branches (Figure 5B). Cells in State 9 (lower isolated cluster) represented the earliest progenitor-like population, while cells in States 2 and 3 occupying the distal right arm exhibited the highest pseudotime values, suggesting a terminally differentiated transcriptional state.

Projection of the five astrocyte subpopulations onto the trajectory revealed a clear subpopulation-specific distribution pattern (Figure 5C). Ast_Slc7a10 and Ast_Son cells were enriched along the early-to-mid pseudotime continuum, consistent with a more primitive or homeostatic identity. In contrast, Ast_S100a6 cells predominantly occupied the terminal branches, particularly the distal arm, supporting a differentiated reactive phenotype. Ast_Serpinf1 and Ast_Tpt1 were distributed at intermediate nodes and branch points, suggesting transitional states bridging homeostatic and reactive programs.

Comparative analysis between the Con and MR groups revealed notable group-specific differences in trajectory occupancy (Figure 5C,D). In the Con group, cells were relatively evenly distributed across the main trajectory axis, whereas MR group cells showed a pronounced accumulation in the terminal branches, particularly along the upper-right arm enriched for Ast_Son and Ast_S100a6. Furthermore, the MR group exhibited a marked depletion of cells at the early pseudotime nodes, implying an accelerated or dysregulated state transition away from the homeostatic ground state. These findings suggest that microwave exposure drives directional astrocyte state progression toward reactive and stress-associated transcriptional endpoints.

### 3.5. Microwave Radiation Remodels Intercellular Communication Networks in the Hippocampus

To systematically characterize the cell–cell communication landscape and its alterations after microwave exposure, we applied CellChat to infer ligand-receptor- mediated intercellular signaling across eight major brain cell types: microglia, oligodendrocytes, OPCs, astrocytes, endothelial cells, pericytes, neurons, and ependymal cells.

**Figure 6:**
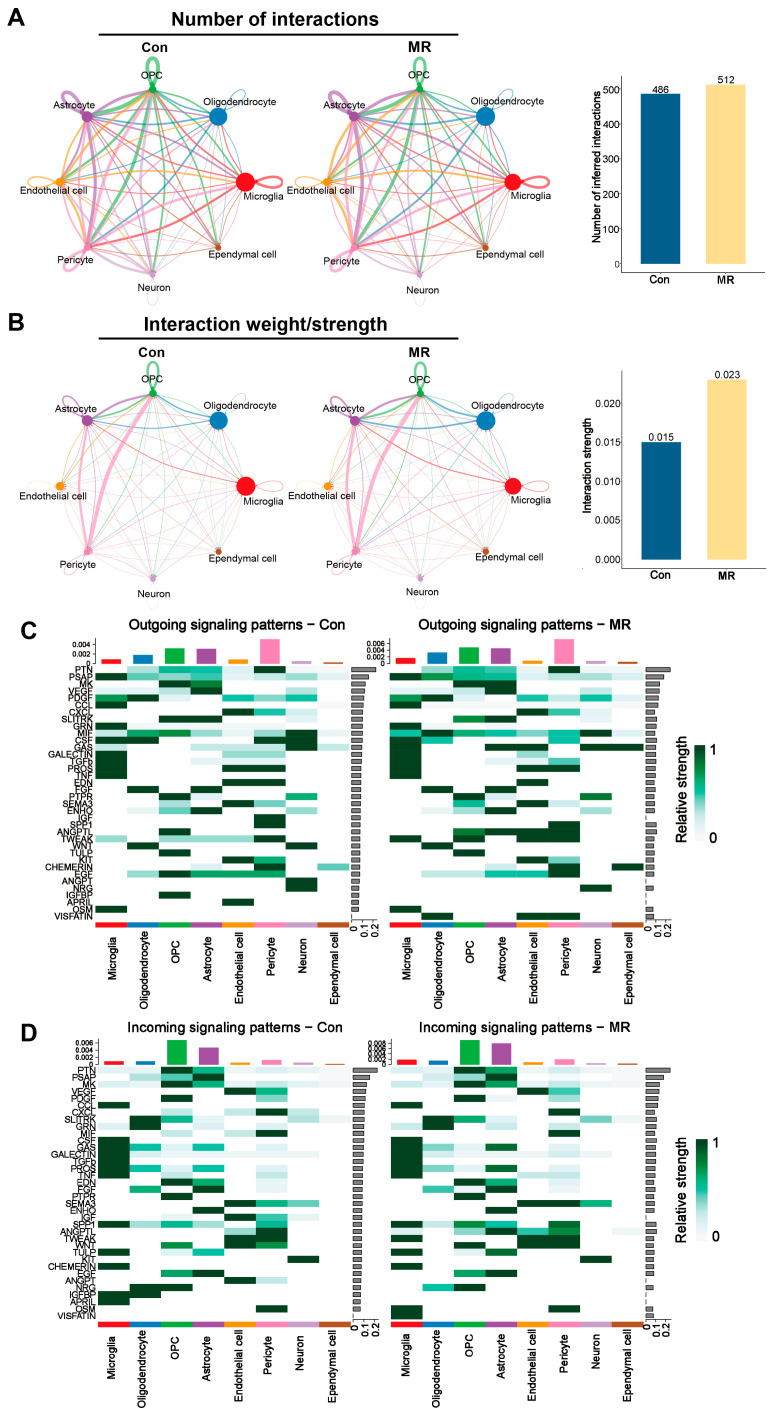
CellChat-based cell–cell communication analysis comparing Con and MR groups. (**A**) Chord diagrams depicting the number of inferred ligand-receptor interactions among eight brain cell types in the Con and MR groups (**left**), with a bar chart summarizing the total interaction counts (Con: 486; MR: 512) (**right**); (**B**) Chord diagrams depicting interaction weight/strength in the Con and MR groups (**left**), with a bar chart summarizing mean interaction strength (Con: 0.015; MR: 0.023) (**right**); (**C**) Heatmaps showing relative outgoing signaling strength for each signaling pathway across cell types in the Con (**left**) and MR (**right**) groups. Color intensity indicates relative signaling strength; (**D**) Heatmaps showing relative incoming signaling strength for each signaling pathway across cell types in the Con (**left**) and MR (**right**) groups.

Global communication landscape. Comparison of the total number of inferred interactions revealed a modest but consistent increase in the MR group (512 interactions) relative to the control group (486 interactions) (Figure 6A). More strikingly, the overall interaction strength was substantially elevated in the MR group (0.023) compared to controls (0.015), representing an approximately 53% increase in communication weight (Figure 6B). Chord diagram visualization of the intercellular communication networks revealed that microglia and astrocytes maintained prominent signaling hubs in both conditions, yet the thickness and density of communication edges. In the MR group, the edge thickness connecting astrocytes, OPCs, and endothelial cells was increased relative to the Con group, indicating a broad amplification of glial and neurovascular crosstalk after microwave exposure.

In the MR group, astrocyte outgoing signaling was increased in four pathways: angiopoietin-like protein (ANGPTL), growth arrest-specific (GAS), macrophage migration inhibitory factor (MIF), and midkine (MK), while two pathways, epidermal growth factor (EGF) and tumor necrosis factor-related weak inducer of apoptosis (TWEAK) showed reduced outgoing signal strength (Figure 6C). The ANGPTL family of secreted proteins has been reported to participate in vascular remodeling and inflammatory regulation in the central nervous system [44], and its upregulation suggests that astrocytes may increase ANGPTL ligand secretion toward endothelial cells and pericytes following radiation exposure. The GAS pathway, whose primary ligand GAS6 signals through AXL and MERTK receptors, has been implicated in the regulation of phagocytic activity and reactive astrogliosis [45]; enhanced outgoing GAS signaling may therefore reflect increased GAS6 secretion by astrocytes acting on neighboring cell types. Upregulation of MIF outgoing signaling is consistent with prior reports that MIF is released by activated astrocytes under stress conditions and contributes to the maintenance of neuroinflammatory states [46]. Increased MK outgoing signaling suggests enhanced astrocyte secretion of midkine, a pleiotropic growth factor with reported roles in both neuroprotection and neuroinflammation depending on context [47]. Conversely, reduced EGF outgoing signaling indicates a possible decrease in astrocyte-derived EGF family ligand secretion, which under homeostatic conditions supports neuronal survival and oligodendrocyte precursor differentiation [48]. The reduction in TWEAK outgoing signaling may reflect altered astrocyte participation in TNF superfamily mediated inflammatory regulation [49].

Concurrently, astrocyte incoming signaling was increased for five pathways: tubby-like protein (TULP), ANGPTL, secreted phosphoprotein 1 (SPP1), protein S (PROS), and GAS (Figure 6D). Enhanced TULP incoming signaling suggests that astrocytes receive increased phagocytic recruitment signals. SPP1 (osteopontin) is predominantly secreted by activated microglia in the context of neuroinflammation [50]. Upregulation of SPP1 incoming signaling indicates that astrocytes receive increased osteopontin-mediated signals from other cell populations following radiation. The concurrent upregulation of ANGPTL and GAS in both outgoing and incoming astrocyte signals suggests the potential existence of autocrine signaling loops for these two pathways.

Taken together, these findings indicate that microwave radiation is associated with a broad remodeling of astrocyte intercellular communication, characterized by increased activity of several inflammatory and phagocytosis-related signaling pathways alongside reduced activity of pathways associated with trophic support.

## 4. Discussion

The hippocampus is considered to be sensitive to MR and is essential for cognitive function. This study designed a Go/No-go task to evaluate water reward memory stimulated by sounds of different frequencies in mice. Additionally, Y-maze and NOR tests were used to evaluate hippocampus-dependent working memory. The findings are consistent with those of previous studies, further confirming MR-induced hippocampal-dependent cognitive impairment in mice.

To investigate cell-type-specific transcriptional alterations in MR-induced hippocampal dysfunction, we performed scRNA-seq to identify different neural cell types and their unique transcriptional features in the mouse hippocampus. Among 94, 088 cells profiled, eight cell types were identified. Sub-clustering analysis identified five transcriptionally distinct astrocyte subpopulations, consistent with emerging evidence from single-cell studies demonstrating that brain astrocytes are not a homogeneous population but comprise multiple functionally specialized subtypes [51,52]. The increase in Ast_S100a6 and Ast_Son proportions and the decrease in Ast_Serpinf1 in the radiation group suggest a compositional shift from homeostatic toward reactive astrocyte states. Ast_S100a6, characterized by high expression of *S100a6*, *Igfbp5*, and *Gfap*, resembles previously described reactive astrocyte signatures associated with neuroinflammatory conditions [53,54]. The reduction in Ast_Serpinf1, a subpopulation expressing neuroprotective genes including *Serpinf1*, *Id1*, and *Id3*, raises the possibility that radiation exposure may diminish a neuroprotective astrocyte state, though this requires direct functional validation.

Pseudotime analysis revealed that radiation-exposed astrocytes were preferentially distributed toward terminal reactive states, with relative depletion at early homeostatic nodes. This pattern is broadly consistent with the concept of reactive astrogliosis, a well-characterized response of astrocytes to CNS injury and stress [55]. The depletion of cells at early pseudotime positions in the MR group may reflect a shift away from homeostatic astrocyte maintenance programs, though it should be noted that pseudotime ordering represents a transcriptional continuum inferred computationally and does not directly capture real-time cellular dynamics. Whether the observed trajectory shifts reflect active state transitions, selective survival, or altered cell proportions cannot be determined from the present cross-sectional data alone.

The differential responses observed among astrocyte subtypes following microwave exposure can be attributed to their intrinsic molecular and functional heterogeneity. Astrocytes are not a homogeneous cell population; rather, they exhibit marked divergence in gene expression profiles, membrane biophysical properties, metabolic activity, and stress-response capacity depending on their regional identity and developmental origin. First, astrocyte subtypes differ in their reliance on oxidative phosphorylation versus glycolysis. Astrocytes with higher mitochondrial density and oxidative metabolic rates are expected to be more vulnerable to microwave-induced disruption of mitochondrial membrane potential and the consequent elevation of reactive oxygen species (ROS), thereby crossing the threshold for oxidative injury more readily [12,56]. Second, the transcriptional programs governing reactive astrogliosis are themselves subtype-dependent. The same injurious stimulus can drive distinct ranscriptional states depending on the intrinsic properties of the astrocyte subtype in question, meaning that identical microwave doses may produce divergent functional outcomes across subtypes [57]. Collectively, these factors indicate that the observed heterogeneity in astrocyte subtype responses to microwave exposure reflects the convergence of subtype-specific molecular machinery governing stress sensing, signal transduction, and cellular adaptation.

To place our findings in a broader context, it is instructive to compare the astrocyte subtype responses observed here with those reported in sequencing studies of other oxidative stress-associated conditions. In models of cerebral ischemia–reperfusion, astrocytes exhibit a rapid upregulation of reactive markers such as *Lcn2* and *Serpina3n*, with a prominent shift toward neuroinflammatory and neurotoxic reactive phenotypes [53]. Similarly, in traumatic brain injury, single-cell analyses have identified an expansion of interferon-responsive and proliferative astrocyte clusters, accompanied by loss of neurotrophic factor expression [58]. Our observation that Ast_S100a6, a subpopulation enriched for calcium signaling and inflammatory pathway genes, expands selectively under microwave exposure aligns with this general paradigm. However, the pronounced and specific depletion of Ast_Serpinf1, a neuroprotective subtype, appears to be a more distinctive feature of microwave-induced stress, possibly reflecting the unique combination of thermal and non-thermal effects that microwaves exert on cellular membranes and ion channels. This comparative perspective highlights both the convergent and divergent features of astrocyte responses to different oxidative insults.

CellChat analysis revealed an overall increase in the number and strength of inferred intercellular interactions in the radiation group, with astrocytes showing notable changes in both outgoing and incoming signaling. The upregulation of MIF outgoing signaling is consistent with established evidence that MIF functions as a pro-inflammatory mediator released by activated glial cells. The upregulation of SPP1 incoming signaling, given that SPP1 is predominantly produced by activated microglia [50], suggests a potential microglia-to-astrocyte signaling relay following radiation, indicating that astrocyte responses may be partly driven by upstream microglial activation. Conversely, the reduction in EGF and TWEAK outgoing signaling suggests decreased astrocyte-derived trophic support under radiation conditions, as EGF family signaling has established roles in supporting neuronal survival and oligodendrocyte differentiation [48]. The concurrent upregulation of ANGPTL and GAS in both outgoing and incoming signals raises the possibility of autocrine signaling loops, though direct experimental validation was not performed in the present study.

An additional and underappreciated dimension of microwave-related health risks concerns the use of microwave-based heating systems in certain electronic cigarette (e-cigarette) models, sometimes referred to as “microwave atomizers”. Unlike conventional resistive heating coils, microwave atomizers use electromagnetic energy to heat and vaporize e-liquid, which may expose users not only to the inhaled chemical toxicants but also to localized microwave radiation in proximity to the oronasal cavity and, potentially, the brain [59]. The combined impact of inhaled oxidative chemicals (e.g., reactive aldehydes, free radicals) and microwave-induced thermal/non-thermal stress could synergistically amplify astrocyte reactivity, accelerating the transition from homeostatic to neuroinflammatory or neurotoxic phenotypes. Although the microwave intensities and frequencies involved in e-cigarette devices differ from those used in our experimental paradigm, the underlying cellular stress pathways may overlap considerably, particularly oxidative stress and TRP channel activation [60]. Future studies specifically examining the neurobiological consequences of microwave-atomizer e-cigarette use, with a focus on astrocyte function and cognitive outcomes, are warranted.

Several limitations of the present study should be acknowledged. First, our experiments were conducted at a single microwave frequency (2856 MHz) and a single intensity, which precludes the assessment of dose–response relationships between microwave parameters and biological effects. Second, pseudotime trajectories represent computationally inferred transcriptional continua and do not directly capture the temporal dynamics of astrocyte state transitions in vivo. Third, the CellChat-inferred intercellular interactions are based on ligand–receptor co-expression and have not been validated by independent biochemical or functional assays; autocrine signaling loops suggested by ANGPTL and GAS co-upregulation in particular require direct experimental confirmation. Future studies incorporating multi-intensity exposure paradigms, lineage-tracing approaches, and functional validation will be essential to build upon the framework established here.

## 5. Conclusions

This study provides single-cell resolution evidence that microwave exposure is associated with astrocyte subpopulation remodeling, directional state transitions toward reactive phenotypes, and altered intercellular communication in the hippocampus. These findings contribute to a better understanding of the cellular basis of microwave radiation-associated hippocampal dysfunction and highlight astrocytes as a potentially relevant cellular target in this context. Taken together, these cellular and molecular alterations provide a mechanistic framework for explaining the hippocampal-dependent cognitive deficits induced by microwave radiation and lay the foundation for the development of targeted therapeutic strategies to mitigate the adverse neurological effects of microwave exposure.

## 6. Future Perspectives

The present findings suggest several directions for future investigation. First, longitudinal single-cell profiling at multiple post-exposure time points, combined with systematic dose–response experiments across a range of specific absorption rates, would clarify the temporal dynamics and threshold characteristics of microwave radiation-induced astrocyte subtype remodeling. Second, cell-type-specific genetic manipulation tools should be employed to establish the causal contributions of individual astrocyte subtypes to hippocampus-dependent cognitive dysfunction. Third, integration of spatial transcriptomics would enable mapping of reactive astrocyte distributions to specific hippocampal subfields, yielding greater anatomical resolution. Finally, validation in human iPSC-derived astrocyte models and identification of circulating biomarkers of astrocyte reactivity would facilitate translation of the present findings toward human exposure risk assessment and potential therapeutic targeting of reactive astrogliosis.

## Data Availability

The original contributions presented in this study are included in the article. Further inquiries can be directed to the corresponding authors.

## Abbreviations

The following abbreviations are used in this manuscript:

scRNA-seq	Single-cell RNA sequencing
MR	Microwave radiation
CNS	Central nervous system
SPF	Specific pathogen-free
PCA	Principal component analysis
OPC	Oligodendrocyte progenitor cell
NOR	Novel object recognition
UMAP	Uniform Manifold Approximation and Projection
